# Effects of experimental conditions on the morphologies, structures and growth modes of pulsed laser-deposited CdS nanoneedles

**DOI:** 10.1186/1556-276X-9-91

**Published:** 2014-02-22

**Authors:** Hui Li, Li Chen, Yu Zhao, Xujun Liu, Leilei Guan, Jian Sun, Jiada Wu, Ning Xu

**Affiliations:** 1 Key Laboratory for Advanced Photonic Materials and Devices, Department of Optical Science and Engineering, Fudan University, Shanghai 200433, People’s Republic of China

**Keywords:** CdS nanoneedles, Substrate temperature, Laser pulse energy, Growth mode, 61.46.-w, 61.46.Km, 68.37.Lp

## Abstract

CdS nanoneedles with different morphologies, structures, and growth modes have been grown on Ni-coated Si(100) surface under different experimental conditions by pulsed laser deposition method. The effects of catalyst layer, substrate temperature, and laser pulse energy on the growth of the CdS nanoneedles were studied in detail. It was confirmed that the formation of the molten catalyst spheres is the key to the nucleation of the CdS nanoneedles by observing the morphologies of the Ni catalyst thin films annealed at different substrate temperatures. Both the substrate temperature and laser pulse energy strongly affected the growth modes of the CdS nanoneedles. The secondary growth of the smaller nanoneedles on the top of the main nanoneedles was found at appropriate conditions. A group of more completed pictures of the growth modes of the CdS nanoneedles were presented.

## Background

Nowadays, semiconductor nanomaterials like nanowires, nanorods, and nanotubes, have aroused great interest in material science and applications owing to their unique characteristics different from film or bulk materials. CdS, as a direct bandgap (2.4 eV) II-VI compound semiconductor, has good optical and electrical properties, which give it potential applications in light-emitting diodes, light sensors, photocatalysts, windows of thin film solar cells as well as absorbers and electrodes of hybrid solar cells [1-7]. Compared to CdS thin films, the CdS nanostructures such as nanoparticles, nanowires, and nanoneedles have higher optoelectronic sensitivities and efficiencies for these devices due to their large surface areas and possible quantum confinement effects [4-7]. There have been many methods for preparing CdS nanowires like electrochemical deposition [8,9], solution process [10,11], chemical and physical vapor deposition, etc. Among the methods, pulsed laser deposition (PLD) is a simple and efficient way to synthesize multicomponent compounds such as II-VI semiconductors [12-14]. It is easy to control the growth rate and avoid materials from pollution as a result of the adjustable frequency of pulsed laser and the good directivity of laser-ablated plasma [13,14]. In our previous work, the CdS nanoneedles have been grown successfully using the PLD method [15] and the growth modes of vapor-liquid-solid (VLS) and vapor-solid (VS) have been suggested [15,16]. In this article, the effects of the substrate temperature and the laser pulse energy on the growth of CdS nanoneedles were studied in detail. Both the VLS and VS growth modes of CdS nanoneedles were further confirmed experimentally. The transformation from VLS to VS growth modes along with the growth of the CdS nanoneedles was discussed.

## Methods

The CdS nanoneedles were deposited on Si(100) substrates using Ni as catalysts by a PLD method. The experimental setup mainly consists of a Nd:YAG laser with a wavelength of 532 nm and a deposition chamber with rotating multitargets and a base pressure of 10^-3^ Pa. High-purity Ni and hot-pressed CdS targets (purchased from Beijing Founde Star Science & Technology Co., Ltd.) with diameter and thickness of 1.5 and 0.5 cm, respectively, were used as sources of precursors of Ni catalyst layer and CdS nanoneedles. Prior to the deposition, substrates were ultrasonically cleaned in acetone and ethanol, etched in HF solution and rinsed in deionized water, successively. To prepare the CdS nanoneedles, there were two steps involved. Firstly, Ni catalysts were deposited on the substrates by PLD with a laser pulse energy of 50 mJ and a repetition rate of 5 Hz for 10 min (without substrate heating). Secondly, the CdS nanoneedles were grown by PLD at different substrate temperatures of 200°C to 500°C, different laser pulse energy of 50 to 80 mJ and a repetition rate of 10 Hz for 30 min. In order to understand the growth mechanism of the CdS nanoneedles, the morphologies of the prepared Ni catalyst-covered substrates were observed after annealing 5 min at the different substrate temperatures of 200°C to 500°C. The morphology of all the samples was examined by field emission scanning electron microscopy (FESEM) and transmission electron microscopy (TEM). The crystalline structures of the CdS nanoneedles were characterized by selected area electron diffraction (SAED) and high-resolution transmission electron microscopy (HRTEM). The composition of the CdS nanoneedles was analyzed by energy-dispersive spectroscopy (EDS) fitted on the TEM.

## Results and discussion

**Figure 1:**
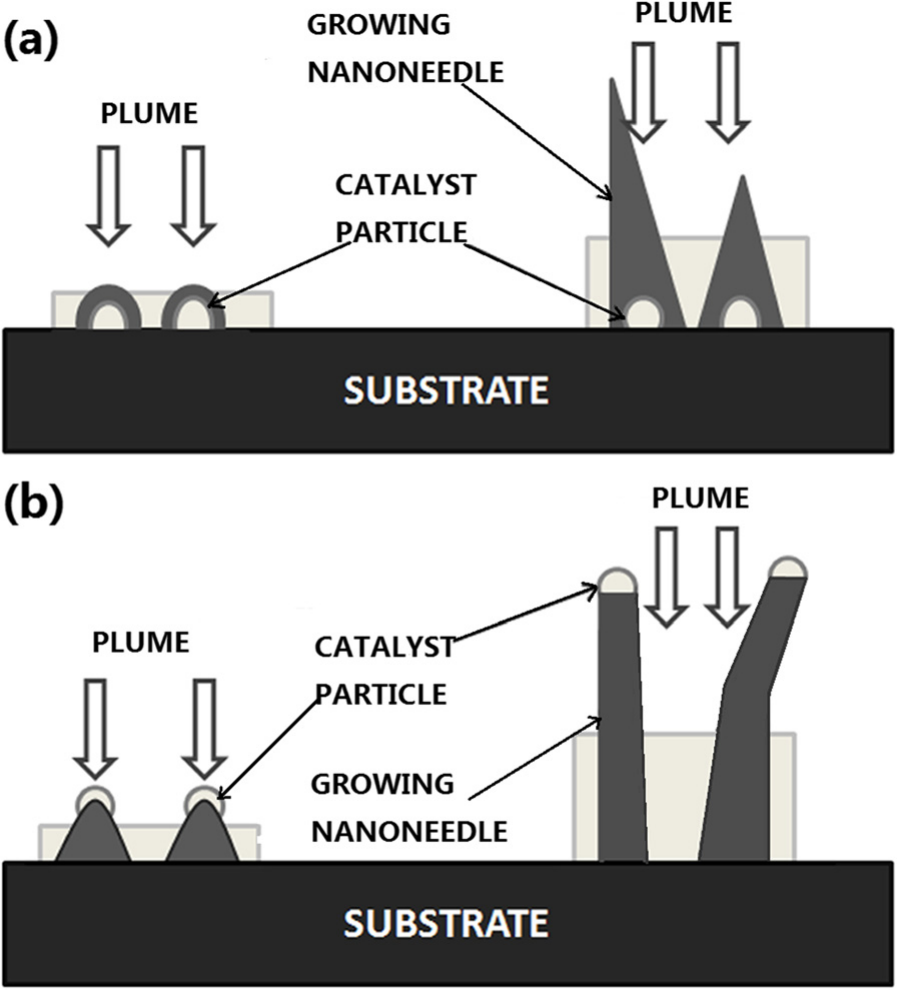
Growth models for CdS nanoneedles of (a) VS and (b) VLS modes.

It has been suggested that the CdS nanoneedles grown by PLD have two main growth modes of VS and VLS (as shown in Figure 1) [15-18]. For the both growth modes, catalyst plays the role of promoting the formation of the crystal nucleus. In the VS growth mode [15,16], the substrate temperature usually is not much high, and the catalyst grains are stable on the substrates. After the nuclei of the CdS nanoneedles are formed on the catalyst grains, the laser-ablated plasma will deposit directly on them, and the CdS nanoneedles can grow out from the background crystallites with priority if they are higher enough (see Figure 1a). In the VLS mode [17,18], the substrate temperature usually is higher, and the catalyst grains are unstable on the substrates. The CdS nucleation would firstly occur at the bottom of the catalyst particles; then, the CdS nuclei push up the catalyst, and the catalyst-leading nanoneedles are eventually formed, as shown in Figure 1b. Because of the instability of catalyst pellets, the nanoneedles were usually crooked.

**Figure 2:**
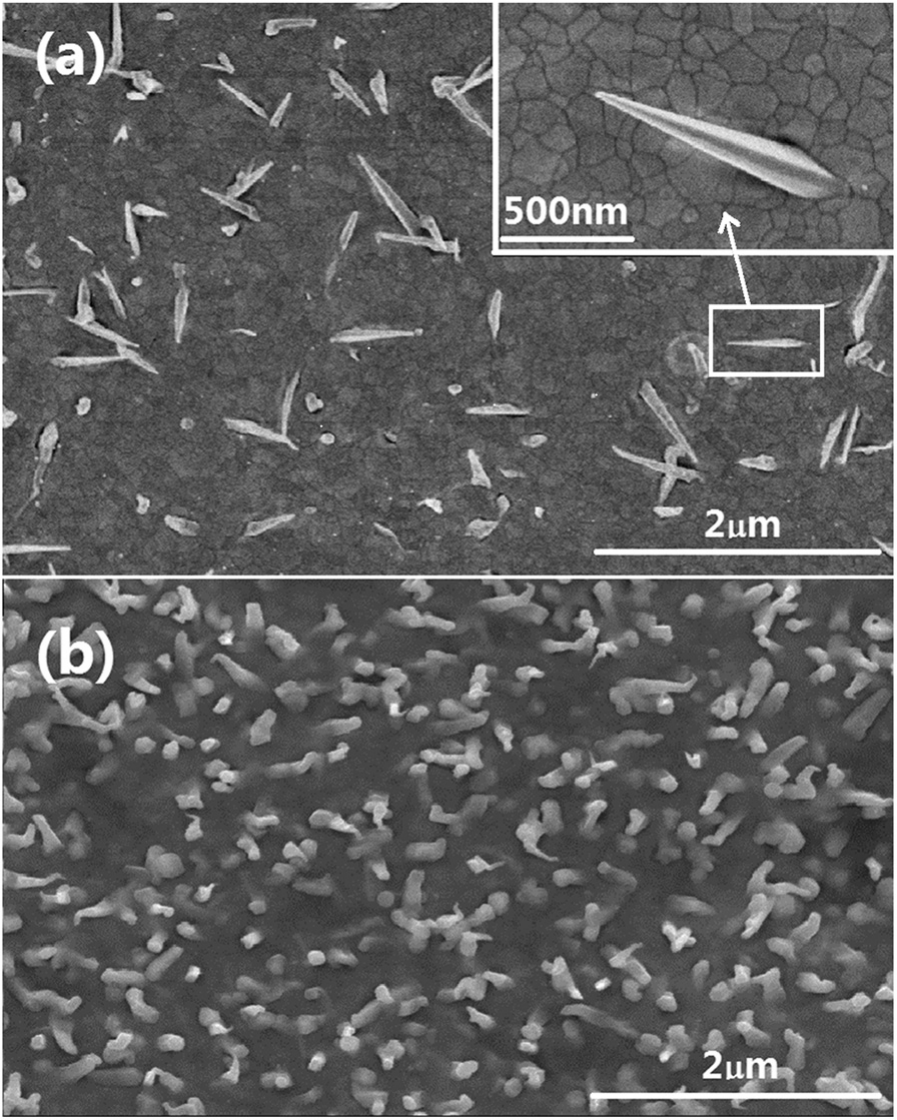
**FESEM images of CdS films grown on Ni-covered Si(100).** At the substrate temperatures of **(a)** 400°C and **(b)** 450°C. The samples were prepared under the same laser pulse energy of 50 mJ. The deposition time, pulse energy, and frequency of catalyst-Ni were 15 min, 50 mJ, and 5Hz, respectively.

The effects of the substrate temperature on the growth of the CdS nanoneedles were examined. When the substrate temperature was changed by the step of 50°C and kept other conditions (a laser pulse energy of 50 mJ, a repetition rate of 10 Hz, a deposition duration of 30 min, Ni layers deposited at 50 mJ, 5 Hz, and 15 min) unchanged, the density of nanoneedles increased higher from zero at a substrate temperature of 200°C to about 4 × 10^8^ cm^-2^ at 400°C and even 2 × 10^9^ cm^-2^ at 450°C; after that, it declined rapidly until the morphology became flat at a substrate temperature of 500°C. The morphology of single nanoneedles prepared at a substrate temperature of 400°C is straight with the average middle diameter and length of 50 and 800 nm, respectively, as shown in Figure 2a. The growth mechanism is typically VS mode, in which the plasma produced by laser ablation directly deposits on the crystal nucleus and the intact nanoneedles are formed. When the substrate temperature was raised to 450°C, the nanoneedles become bent and have catalyst balls on the tops, which indicates the catalyst-leading VLS growth mode of the CdS nanoneedles (see Figure 2b).

**Figure 3:**
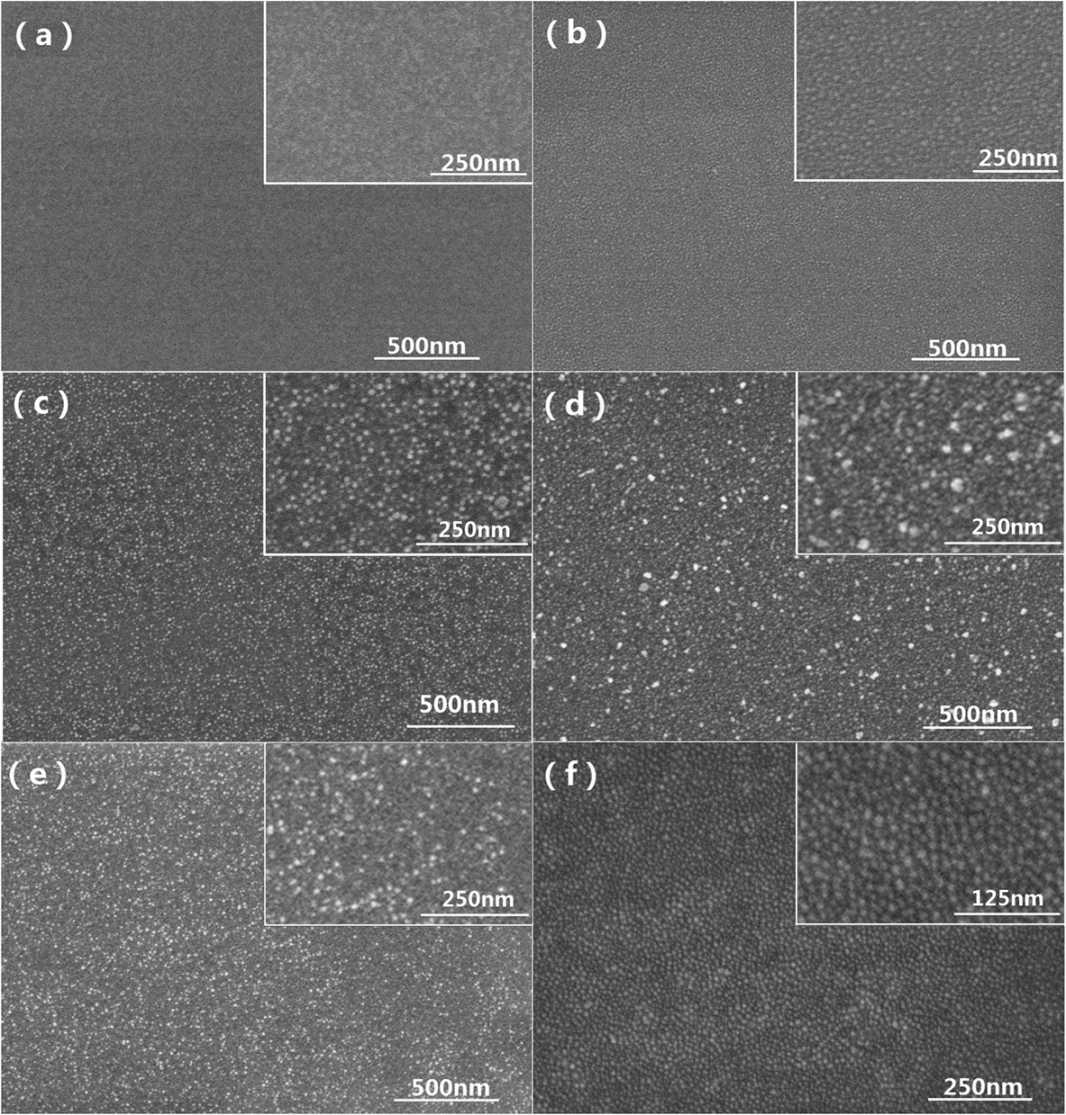
**FESEM images of Ni layers on Si(100) after annealing at different temperatures. (a)** 200°C, **(b)** 300°C, **(c)** 400°C, **(d)** 450°C, **(e)** 475°C, and **(f)** 500°C. The deposition time, laser pulse energy, and frequency of Ni layers were 10 min, 50 mJ, and 5Hz, respectively.

**Figure 4:**
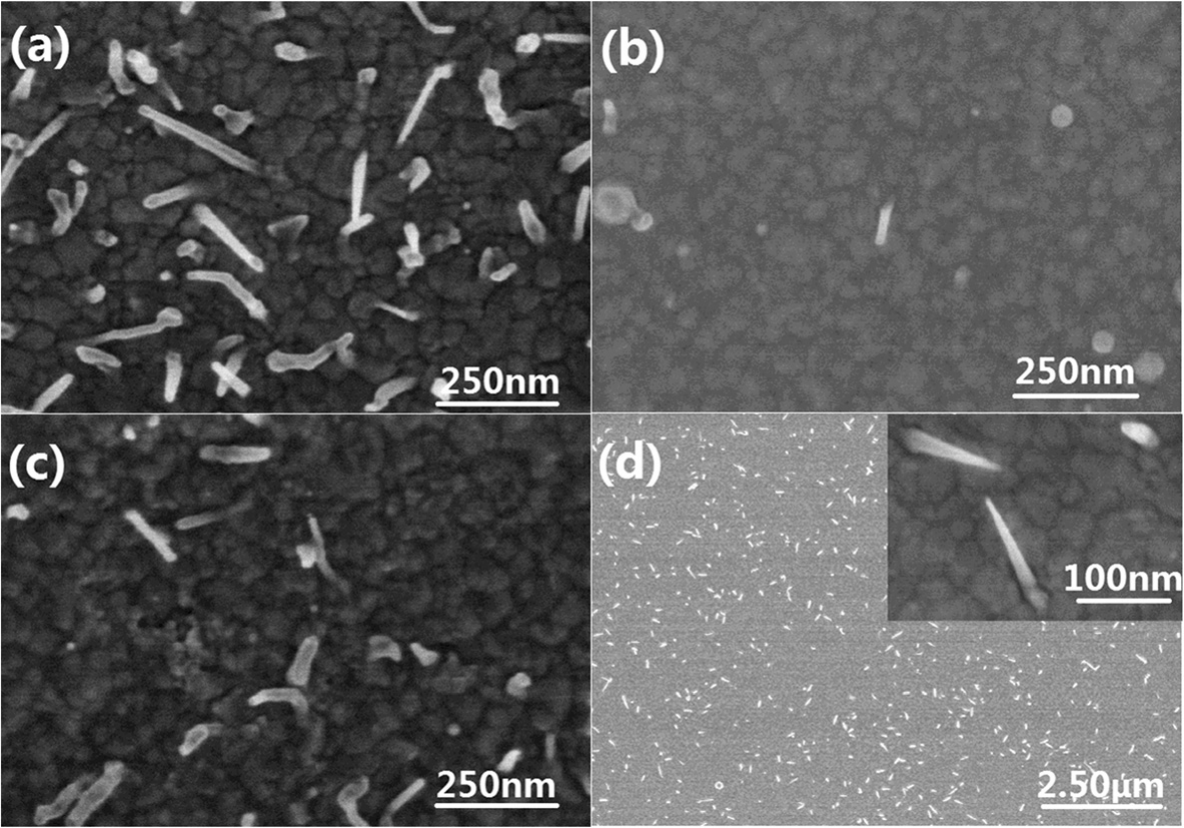
**FESEM images of CdS films grown on Ni-covered Si(100) substrate under different laser pulse energy. (a)** 50 mJ, **(b)** 60 mJ, **(c)** 70 mJ, and **(d)** 80 mJ. The samples were prepared at the temperature of 475°C, and the deposition time, laser pulse energy, and frequency of catalyst-Ni were 10 min, 50 mJ, and 5 Hz, respectively.

In the nucleation of the CdS nanoneedles, it has been thought that the laser-ablated precursors firstly deposit on the molten catalyst spheres or migrate to them from the substrate, then dissolve into the molten catalyst pellets and separated out around the pellets after saturation. So, the formation of the molten catalyst spheres is the key to the nucleation of the CdS nanoneedles. The morphologies of the Ni catalyst thin films annealed at different substrate temperatures for 5 min were shown in Figure 3. It is apparent in Figure 3a,b,c that the Ni thin films gradually melted and the Ni spheres began to form with the increase of the temperature from 200°C to 400°C. In Figure 3a, the Ni film annealed at 200°C substrate temperature has a contiguous flat surface, indicating that it does not melt before and thus cannot lead to the nucleation of the CdS nanoneedles. That is why the CdS nanoneedles could not grow out under such circumstance. At 300°C substrate temperature, the film surface appears some fluctuations, indicating that the Ni film starts to melt (Figure 3b). Until the 400°C substrate temperature, the densely distributed spheres with several nanometers emerge, revealing that the Ni film melts into separated liquid spheres (Figure 3c). In this case, the molten Ni spheres can play the role of promoting the nucleation of the CdS nanoneedles. In Figure 3d, it can be seen that the whole thin film has molten into dense spheres at 450°C substrate temperature and some big grains with tens of nanometers are formed. This situation corresponds to that of Figure 2b, in which dense CdS nanoneedles were grown in accordance with the VLS mode. However, the molten Ni spheres become smaller and more sparse at the 475°C substrate temperature. The morphologies of the Ni thin films are very sensitive to the substrate temperatures at around 450°C to 475°C. In these situations, the CdS nanoneedles could be grown according to the VLS and VS modes at a laser pulse energy of 50 and 80 mJ, respectively, and are sometimes sparse as shown in Figure 4. When the substrate temperature rose to about 500°C, the molten Ni thin film becomes undulating in morphology again and no obvious spheres could be found (Figure 3f). In this situation, CdS nanoneedles also cannot grow out. The above morphologies of the Ni catalyst thin films annealed at 200°C to 500°C substrate temperatures are basically in line with the growth situations of the CdS nanoneedles.

In order to better understand the effects of experimental conditions on the growth mechanism of the CdS nanoneedles, the laser pulse energy was changed in a series of experiments for preparation of CdS nanoneedles. In the experiments, the conditions of Ni deposition (50 mJ, 5 Hz, and 10 min) and the substrate temperature of CdS deposition (475°C) were kept unchanged, and the laser pulse energy was set from 50 to 80 mJ by every step of 10 mJ. The influence of the laser energy on the growth of the CdS nanoneedles is shown in Figure 4. In Figure 4, as the laser pulse energy is 50 mJ, there are many crooked and straight nanoneedles grown on the polycrystalline background with catalyst balls on the tops, which accords with the VLS growth mode. When the pulse energy increases to 60 mJ, almost no nanoneedles grow on the polycrystalline background film. Continuing to increase the laser pulse energy to 70 mJ, some nanoneedles grow out again, but they have some bent and poor shapes without catalyst balls on the tops. If the laser pulse energy is increased to 80 mJ, not only the size and density of the as-grown nanoneedles increase but also they have intact nanoneedle shapes, which is the typical VS growth mode. From Figure 4a,b,c,d, it could be found that the growth modes of the CdS nanoneedles change from the VLS mode to the VS mode with the increase of the laser pulse energy from 50 to 80 mJ, which reveals that the laser pulse energy strongly affected the growth of the CdS nanoneedles. With the increase of the laser pulse energy, the kinetic energy and density of the laser-ablated plasma increase and the CdS thin films are deposited faster, which would lead to that the incipient CdS nanoneedles are covered by the growing base thin films and the CdS nanoneedles grown in the VLS mode cannot grow out. This may be also related to the sputtering-off effect of the laser-ablated plasma on the catalysts, i.e., that the bombardments of plasma on the tops of the incipient CdS nanoneedles restrain the VLS growth of the CdS nanoneedles. In Figure 4c, the as-grown CdS nanoneedles have no catalyst balls on the tops, which may be due to such plasma bombardment. The growth mode of these CdS nanoneedles may have been converted to the VS mode at certain laser pulse energy (for example, above 70 mJ). In this case, the kinetic energy and density of the laser-ablated plasma will satisfy the VS growth conditions of CdS nanoneedles and make the incipient CdS nanoneedles grow faster without catalyst-leading than the base thin films as shown in Figure 4d.

**Figure 5:**
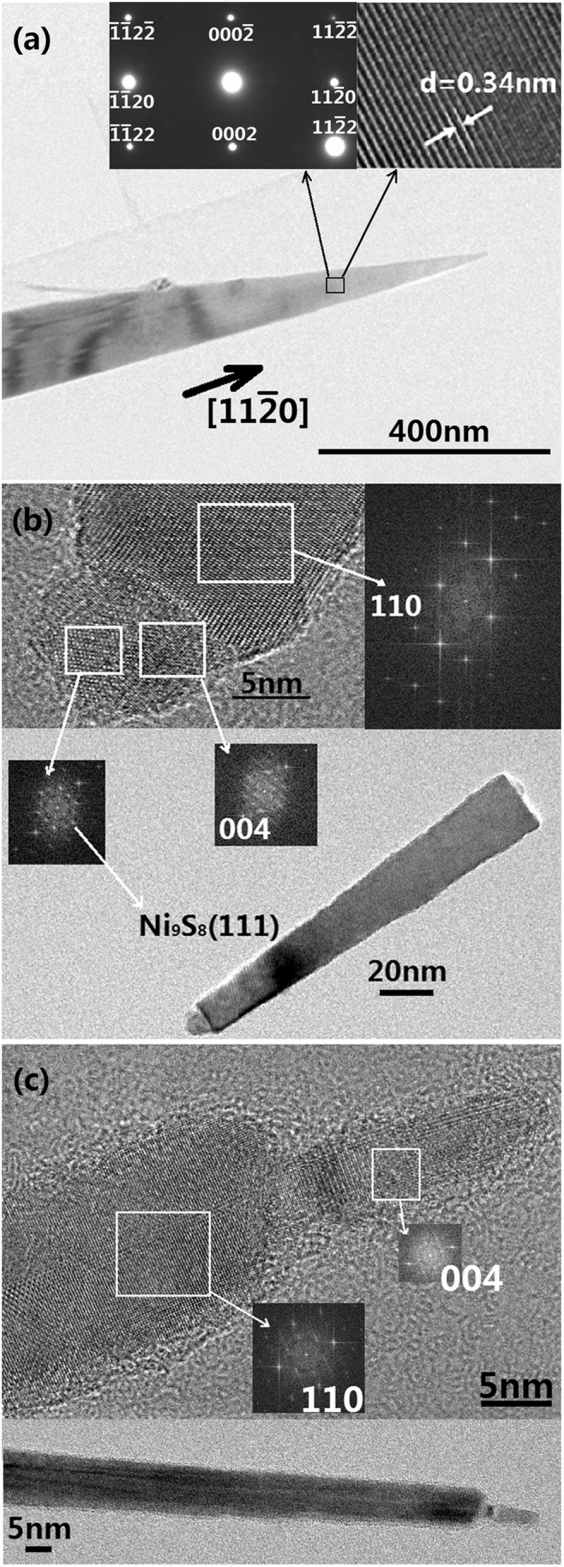
**TEM morphologies, HRTEM images and FFT diagrams.** TEM morphology **(a)** of a CdS nanoneedle grown at the substrate temperature of 400°C (in VS mode), with a SEAD pattern in left upper inset and high-resolution image in right upper inset; **(b and c)** TEM morphologies, HRTEM images, and FFT diagrams (at different locations) of the CdS nanoneedles grown at the 475°C substrate temperature. Panel **(b)** shows a CdS nanoneedle (grown in VLS mode) with a half ball of the mixture of CdS and Ni on the top; panel (c) shows a main CdS nanoneedle (grown in VLS mode) with a secondary CdS nanoneedle (grown in VS mode) on the top.

**Figure 6:**
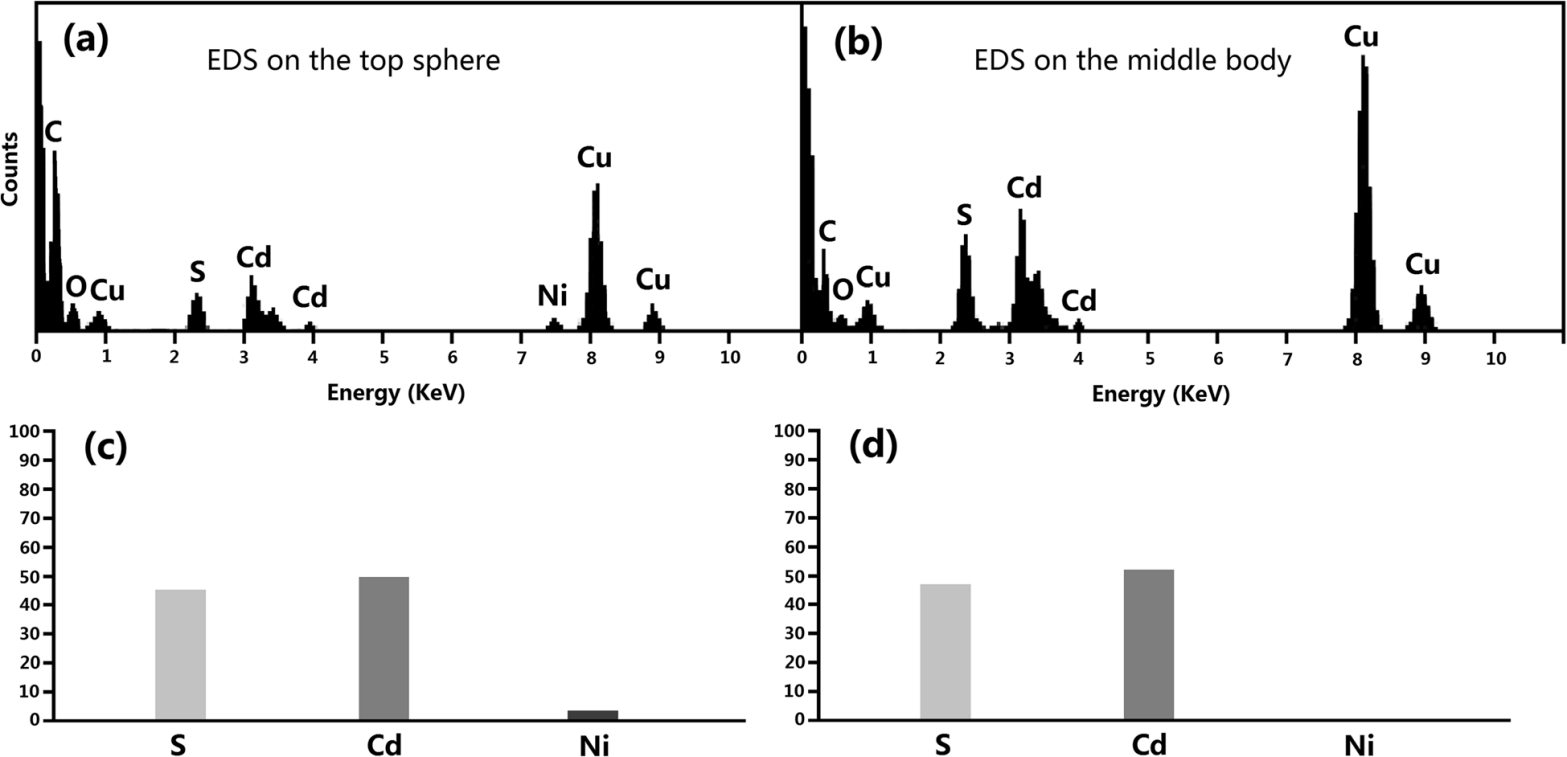
**EDS spectra and the analytical results. (a and b)** EDS spectra at the top and middle positions of a CdS nanoneedle grown at the 475°C substrate temperature (in VLS mode); **(c and d)** the analytical results of the above EDS spectra. Panels **(a)** and **(c)** show the EDS spectrum and its analytical result of the half ball on the top of the CdS nanoneedle (shown in Figure 5b), respectively; panels **(b)** and **(d)** show the EDS spectrum and its analytical result of its main body.

In order to further confirm and comprehend the growth mechanism of the CdS nanoneedles, TEM, HRTEM, and EDS were carried out to observe the morphology, composition, and the structure of the CdS nanoneedles in detail. Details of the CdS nanoneedles grown at a substrate temperature of 400°C (as shown in Figure 2a) were further clarified by TEM (Figure 5a). In Figure 5a, the morphology of a single CdS nanoneedle is regular long taper. No existence of Ni catalyst on the top of the CdS nanoneedle indicates its typical VS growth mode. The SEAD pattern and HRTEM image in right upper inset exhibits that the nanoneedle is single-crystalline CdS with the orientation of $\left(11\overset{¯}{2}0\right)$ perpendicular to the plane of (0002), and the distance between the planes of (0002) was 0.34 nm. The sample shown in Figure 5b was prepared at the temperature of 475°C; the deposition time and the pulse frequency of Ni was 10 min and 5 Hz, respectively. In Figure 5b, a catalyst ball on the top of an as-grown nanoneedle is very apparent. Figure 6 gives EDS spectra at the top and middle positions of the CdS nanoneedle shown in Figure 5b and their analytical results. In Figure 6a,b, the Cu, C, and O should come from the copper grids covered by about 10 nm carbon thin films (supporter of the samples for TEM observation) and the exposure of the samples to air for a long time; therefore they were not included in the analytical results (Figure 6c,d). The EDS analyses on the top and middle positions of the nanoneedle show that the percentages of both Cd and S are approximately equal and those of Ni is about 3.46% on the top and below the detection limit in the middle position (as shown in Figure 6c,d). Because the EDS is only a semi-quantitative analysis tool, its analysis results are usually of some deviation from the actual situation. The existence of Ni only on the top of the nanoneedle illustrates the catalyst-leading growth of the nanoneedles, i.e., the VLS growth mode. The HRTEM of the nanoneedle top was analyzed further by the fast Fourier transform (FFT). From the FFT patterns, the structure of the top can be figured out by calculating the lattice distance. The FFT patterns in the inserts of Figure 5b show that the nanoneedle body is a hexagonal structure of CdS crystal with the (110) direction while the sphere on the top is mixed structures of hexagonal CdS with the (004) and (101) directions and orthorhombic Ni_9_S_8_ with the (111) direction [19-21]. No pure Ni lattices but mixtures of CdS and Ni_*x*_S_1-*x*_ in the top sphere indicates that Cd and S entered the molten catalyst during the CdS nanoneedle growth, and the orthorhombic Ni_9_S_8_ was crystallized in the later cooling process.

**Figure 7:**
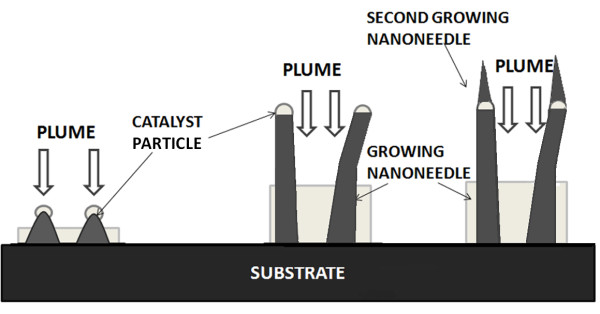
Growth model for the secondary growth of CdS nanoneedle.

In the growth of CdS nanoneedles, an interesting phenomenon was found in the sample prepared at the substrate temperature of 475°C (Figure 5c), which could explain the growth mechanism more. Figure 5c shows that a small nanoneedle grew secondarily on the top of the as-grown main nanoneedle. FFT patterns manifest that the main nanoneedle is hexagonal structure with (110) direction while the secondary nanoneedle is hexagonal in structure with (004) direction and without catalysts leading, which indicates that the growth mode of the small nanoneedle on the top turns from VLS to VS with the change in the growth direction. This may be due to that the temperature of the Ni sphere on the top of the growing CdS nanoneedle decreases to satisfy the VS growth conditions as the CdS nanoneedle grow to a certain length. The growth of the small CdS nanoneedle on the top of the main nanoneedle is called the secondary growth mode as shown in Figure 7.

## Conclusions

In conclusion, the substrate temperature and the pulse laser energy affect the growth mode of the CdS nanoneedles, but the influenced factors are interacted. The formation of the molten catalyst spheres is confirmed to be the key to the nucleation of the CdS nanoneedles by observing the morphologies of the Ni-catalyst thin films annealed at different substrate temperatures. Under the certain conditions, changing the substrate temperature or the pulse laser energy may cause the changes of the growth modes of the CdS nanoneedles. In our experiments, under the same laser energy, the growth mode of the CdS nanoneedles is VS at a substrate temperature of 400°C, but it turns into VLS at a substrate temperature of 450°C. Also, altering the pulse laser energy from 50 to 80 mJ may also change the growth modes of the CdS nanoneedles from VLS to VS. Besides, the secondary growth of the smaller CdS nanoneedles is found on the tops of the main CdS nanoneedles. In secondary growth mode, the main CdS nanoneedles grow in VLS mode with catalysts leading, and the secondary CdS nanoneedles grow in VS mode without catalysts leading due to the decrease of the temperature of the Ni spheres on the tops of the main nanoneedles.

## Abbreviations

EDS: energy-dispersive spectroscopy; FESEM: field emission scanning electron microscopy; FFT: fast Fourier transform; HRTEM: high-resolution transmission electron microscopy; PLD: pulsed laser deposition; SAED: selected area electron diffraction; TEM: transmission electron microscopy; VLS: vapor-liquid-solid; VS: vapor-solid.

## Competing interests

The authors declare that they have no competing interests.

## Authors' contributions

HL and LC designed and carried out the experiments, and wrote the paper. YZ, XL and LG participated in the experiments. JS and JW participated in the design and the discussion of this study. NX conceived and designed the experiments, and revised the paper. All authors read and approved the final manuscript.
